# Growth of Marine Microalgae in Landfill Leachate and Their Ability as Pollutants Removal

**DOI:** 10.21315/tlsr2021.32.2.9

**Published:** 2021-06-29

**Authors:** Asma Liyana Shaari, Siti Norsyuhaila Che Sa, Misni Surif, Noorazah Zolkarnain, Razmah Ghazali

**Affiliations:** 1 Malaysian Palm Oil Board, 6, Persiaran Institusi, Bandar Baru Bangi, 43000 Kajang, Selangor, Malaysia; 2 Malaysia Knowledge Transfer Centre (KTC), Level 1, TORAY-USM, 11800 USM Pulau Pinang, Malaysia

**Keywords:** Chemical Oxygen Demand, Ammonia-Nitrogen, *Chlorella* sp., *Nannochloropsis* sp, Keperluan Oksigen Kimia, Ammonia-Nitrogen, *Chlorella* sp., *Nannochloropsis* sp

## Abstract

Leachate from landfill contains concentrated nutrients that may enter the terrestrial and aquatic environment, including nearby coastal areas. The nutrient contaminants eventually bring harm to marine organisms, including microalgae. This study was performed to investigate the growth of two green microalgal species, *i.e. Chlorella* sp. and *Nannochloropsis* sp. in diluted landfill leachate. Besides, the ability of nutrient removal by these microalgal was also explored from the changes of chemical oxygen demand (COD) and nutrients content. The initial and final concentrations of COD, NH_3_-N, and PO_4_^3−^ in the diluted leachate (5%, 10% and 15%) were measured and the growth patterns of these species were determined by counting the cell numbers for 12 days. Comparison of these microalgae showed that the growth rate of *Nannochloropsis* was significantly higher compared to *Chlorella* in all leachate concentrations. Leachate at 5% enhanced the growth of both microalgae, while leachates at 10% and 15% decreased their growth as early as at the beginning of the test. It is apparent that the less concentrated leachate discharged into seawater would not pose any toxicity to the environment and would not bear adverse effect to microalgae yet could promote their growth. This study also revealed that the microalgae could remediate leachate pollution by its ability of nutrient removal; thus, leading to the potential application in wastewater bioremediation, including industrial waste and palm oil mill effluent.

## Abstract (fr)

Larut lesapan dari tapak pelupusan mengandungi bahan pencemar atau nutrien berkepekatan tinggi yang boleh mencemarkan persekitaran daratan dan akuatik, termasuk kawasan pantai berdekatan. Pada masa ini, pencemaran larut lesapan di kawasan pantai membawa satu kebimbangan yang serius. Bahan pencemar ini boleh membahayakan organisma marin termasuk mikroalga. Kajian ini melaporkan pertumbuhan dua spesis mikroalgal hijau, iaitu *Chlorella* sp. dan *Nannochloropsis* sp. di dalam larut lesapan yang telah dicairkan. Perubahan permintaan oksigen kimia (COD) dan kandungan nutrien, iaitu amonia-nitrogen (NH_3_-N) dan fosfat (PO_4_^3−^) juga dipantau. Corak pertumbuhan mikroalga telah dikenal pasti selepas dikultur di dalam larut lesapan yang telah dicairkan dengan kepekatan 5%, 10% dan 15% selama 12 hari. Bacaan awal COD, NH_3_-N, dan PO_4_^3−^ sebelum kajian dan bacaan akhir COD selepas kajian diukur. Dari segi kepekatan, larut lesapan pada 5% meningkatkan pertumbuhan, sementara larut lesapan pada 10% dan 15% menurunkan pertumbuhan kedua-dua spesis mikroalga seawal permulaan ujian. Perbandingan dari segi spesis mikroalga pula menunjukkan bahawa kadar pertumbuhan *Nannochloropsis* jauh lebih tinggi berbanding *Chlorella* dalam semua kepekatan larut lesapan yang diuji. Walau bagaimanapun, dalam larut lesapan berkepekatan 5%, *Chlorella* menunjukkan keupayaan untuk menyah nutrien berlebihan dengan lebih cekap berbanding *Nannochloropsis*. Secara keseluruhan, kajian ini memberi gambaran bahawa larut lesapan yang berkepekatan rendah di dalam air laut tidak akan menimbulkan kesan buruk kepada alam sekitar dan mikroalga, tetapi sebaliknya dapat meningkatkan pertumbuhan mikroalga tersebut. Kajian ini juga mendedahkan bahawa mikroalga boleh membantu dalam mengurangkan pencemaran oleh larut lesapan disebabkan keupayaannya dalam menyingkirkan lebihan nutrien. Oleh itu, kajian ini merintis kepada potensi penggunaan mikroalga di dalam bidang bioremediasi air sisa, termasuklah untuk sisa industri dan air kumbahan dari kilang minyak sawit.

**Highlights**

- Green microalgae, Nannochloropsis is able to grow better than another green microalgae species, i.e. Chlorella in leachate medium, an alternative growth media for mass cultivation.
- Low concentration leachate that discharged into seawater would not pose any toxicity to the environment and would not bear adverse effect to microalgae yet could promote their growth.
- There is feasibility of using Nannochloropsis and Chlorella for waste water treatment, including industrial waste and palm oil mill effluent because both species exhibited an ability to remove excessive nutrients.

## INTRODUCTION

The landfill is the most common method used in the disposal of municipal solid residues in many developing countries (Al-Wabel *et al*. 2011). The landfilling can lead to harmful emissions of gas and leachate, which contains large amounts of organic and inorganic contaminants (Raghab *et al*. 2013). Owing to financial constraints, landfill usually lacks environmental abatement measures, such as leachate collection system and lining materials (Ismail & Manaf 2013). Therefore, contaminations are inflicted upon the environment, especially in soil, groundwater and surface water, including the ocean. As a result, these contaminations threaten the health of exposed populations and ecosystem (Zhang *et al*. 2010).

As a general rule, wastewater generated in a municipal landfill, namely leachate, is characterised by high values of chemical oxygen demand (COD), pH, NH_3_-N and heavy metals, as well as intense colour and bad odour. According to Baun *et al*. (2000), landfill leachate contains a high concentration of organic matters and inorganics ions, including heavy metal, which can be a source of great concern to the aquatic environment. Leachate from landfill could enter nearby terrestrial area, stream or coastal area and spread a large number of toxins into receiving water area *via* seepage and leakage, creating a major environmental issue. Typically, the existence of high levels of contaminants, such as NH_3_-N in the leachate over a long time leads to the pollution of nearby land and waterways such as rivers, lakes, reservoirs, estuaries and marine waters.

It is well known that microalgae are relatively sensitive to many chemicals and responsive to subtle changes or disturbances, such as the presence of pollutants in their surrounding environment that may not visibly affect other organisms or may only affect other organisms at higher levels of disturbance (Stevenson & Bahls 1999). Thus, microalgae are frequently used in bioassays for the toxicity assessment of different contaminants and pollutants (Cid *et al*. 2012). As indicated by Cremen *et al*. (2007), Malaysian marine environment is naturally home to many microalgal species due to its physical, chemical and biological factors that allow the production of microalgae to a great extent. Therefore, the use of marine microalgae as test species for marine water samples is possible, which usually indicate their ecological relevance. According to Marchao *et al*. (2017), microalgae have already been applied in effluent treatment to remove nitrogen, phosphorus and COD, from different types of effluent. It is, therefore, crucial to assess the toxicity of toxicants, in this case, the landfill leachate, to microalgae, as the pollution may affect the marine ecosystems. Besides, Pham and Bui (2020) also reported that microalgae (*Scenedesmus* sp.) enhance the removal of nutrients and COD from wastewater effluent and the results indicated that the wastewater could be used as a cost-effective growth medium for microalgae.

In this study, the main objective was to determine the effects of three different concentrations of leachate (5%, 10% and 15%) upon the growth of two species, i.e., *Chlorella* sp. and *Nannochloropsis* sp. The *Chlorella* is a simple unicellular green alga, which is easy to cultivate and can be found in both marine and freshwater environment, depending on the species (Hibbert 1981). *Chlorella* is a medium-sized spherical cell with 2 μm–10 μm diameter, depending on the species. There is a prominent pyrenoid within its cup-shaped chloroplast; therefore, it is widely used in various physiological studies. Meanwhile, *Nannochloropsis* is distributed in oceans worldwide. This species is characterised by spherical or slightly ovoid cells with a diameter of 2 μm–5 μm. It has one chloroplast in each cell, without pyrenoid and the absence of chlorophyll b and cellular xanthophylls pigment (Hibbert 1981). The growth patterns in terms of cell density of the algae after 12 days of exposure to diluted leachate were studied. To this deed, measurements of nutrient content in the three concentrations of leachate were carried out before the initiation of the experiment and after 12 days of microalgal cultivation.

## MATERIALS AND METHODS

### Microalgae Species

Two microalgae species used in the study, i.e., *Chlorella* sp. and *Nannochloropsis* sp., were obtained from Fisheries Research Institute, Department of Fisheries, Batu Maung, Pulau Pinang, Malaysia. Both cultures were maintained in the widely used algae medium, Walne’s, also known as Conway (Walne 1970).

### Preparation of Leachate Culture Media and Positive Control Medium

The leachate samples were collected from pre-treated effluent pool at Pulau Burung Landfill Sanitary, Nibong Tebal, Seberang Perai Selatan, Pulau Pinang, Malaysia (5°24′N, 100°24′E). The leachate samples were transported to the laboratory in a cold container at 4°C to minimise biological and chemical changes prior to the experimental use. The samples were filtered immediately after arrived using glass fiber filter Whatman® GF/C (0.45 μm) to retain fine particles from passing through. The samples were then diluted to three different concentrations: 5%, 10% and 15% (50 mL, 100 mL and 150 mL leachate, respectively, in 1000 mL Conway medium) with salinity 30 ppt. The diluted leachates were used as culture media. The pH of culture media was adjusted to 8.0 ± 0.2 before microalgae inoculation.

### Measurement of Nutrients

The COD concentration of diluted leachate samples was measured using photometric method (Hach DR2010), while initial ammonia-nitrogen (NH_3_-N) and phosphate (PO_4_^3−^) concentrations were measured based on Hach Salicylate method and Hach Ascorbic Acid method, respectively using Spectrophotometer (Hach 2800) (Hach Company 2002). Measurements were carried out on triplicate samples prior to initiation of experiment and after 12 days of microalgae cultivation to analyse changes in nutrients concentration before and after inoculation of microalgae. Liquid samples for final nutrient analysis was collected from the culture flask and the microalgae were filtered through Whatman® GF/C (0.45 μm) membrane filters with the aid of a vacuum filtration apparatus. The filtrate was collected for analysis of post-cultivation nutrient. This post-cultivation nutrient analysis was performed to investigate the possibility of nutrient removal by these microalgae. Removal efficiency for those nutrients was calculated by the difference in concentration between the initial day and after 12 days of cultivation. Diluted leachate samples without inoculation of microalgae were prepared as control.

### Preparation of Stock Microalgae

Microalgae stock cultures were prepared in 250 mL Erlenmeyer flasks, containing 200 mL Conway culture medium (Walne 1970). The flasks were seeded with sub-cultured microalgae and they were allowed to reach exponential phase for about four to five days.

### Preparation of Inoculants

The total of 250 mL culture flasks containing various concentrations of leachate as culture medium were inoculated with exponentially growing microalgae from the stock microalgae culture prepared earlier under laminar flow hood. The following formula was used to compute the required volume of stock microalgae culture to be added into the culture flasks to give a desired initial density of 1 × 10^6^ cells/mL (Tendencia *et al*. 2005).

$$\begin{array}{l} \text{Required volume of stock microalgae to be added}=\\ \frac{\left(\text{desired density for culture flask}-\text{existing density in culture flask})\times (\text{vol.}\;\text{of medium culture flask}\right)}{\text{density of stock culture}} \end{array}$$

The required volume of stock culture to be added was then filtered through Whatman® GF/C (0.45 μm) cellulose nitrate membrane filters with the aid of a vacuum filtration apparatus (B-169 Vacum-System, Merck KGaA, Darmstadt, Germany). The filter membranes with concentrated microalgae were rinsed with 100 mL of culture media into the culture flasks to collect the required density of microalgae. Subsequently, another 100 mL of culture media was added into the flasks. Then, each flask was closed with a sterile cotton plug that covered with aluminum foil. Experiments were conducted in triplicate by exposing two species of marine microalgae, *Chlorella* and *Nannochloropsis* to three different concentrations of leachate (5%, 10% and 15%). The growths of both microalgae were monitored for 12 days. Prior to initiation of experiment, all cultures in the culture flasks were acclimated at a constant culture conditions with temperature of 25 ± 2ºC and light intensity of 40–50 μmol photon/m^2^/s in 12:12 h light dark under a white fluorescent lamp, and these parameters were maintained throughout the experiment.

### Measurement of Cell Density and Growth Rate of Microalgae

Microalgae growth was determined by their cell density using the bright line heamacytometer (Improved Neubauer, 0.1 mm deep, Hausser Scientific, USA) at two-day intervals and duplicate counts were performed for each culture flask by light microscope (CME, Leica, Wetzlar, Germany). Growth rate (*r*) during exponential phase was determined using the following equation.

$$r=\frac{ln(N_{t}/N_{0})}{\Delta_{t}}=\frac{{lnN}_{t}-{lnN}_{0}}{\Delta_{t}}$$

where, *r* is the growth rate per day. Δ*_t_* is the length of the time interval (*t*_0_–*t*_1_), *t*_0_ is time at the starts of the exponential phase (day), while *t*_1_ is time at the end of the exponential phase (day). *N*_0_ is the number of microalgae cells at the start of exponential phase (cell/mL), and *N*_t_ is the number of cells at the end (cell/mL) (Wood *et al*. 2005).

### Statistical Analysis

The one-way ANOVA and Duncan’s multiple range tests were performed on the cell density and final concentration of COD and nutrients in cultures to determine significant differences between parameters at *P* < 0.05. The analysis was conducted by SPSS 13.0 software.

## RESULTS

### Characteristics of Leachate Samples

The physical characteristics of diluted leachate can be described as light to dark brown colour with obnoxious and unfavourable odour, whereas the chemical characteristics, i.e., initial COD, NH_3_-N, and PO_4_^3−^, are shown in Table 1.

The results obtained showed that the initial COD reading for 5%, 10%, and 15% diluted leachate are 2500 mg/L, 3500 mg/L, and 4500 mg/L, respectively. These diluted leachates also contained a relatively high NH_3_-N concentration, i.e., 150 mg/L, 250 mg/L and 350 mg/L, respectively. Besides that, the PO_4_^3−^ values are 0.5 mg/L, 1.0 mg/L, and 1.5 mg/L in the respective diluted leachate (Table 1).

### Cell Density and Growth Rate of *Chlorella* and *Nannochloropsis* in Leachates

**Figure 1:**
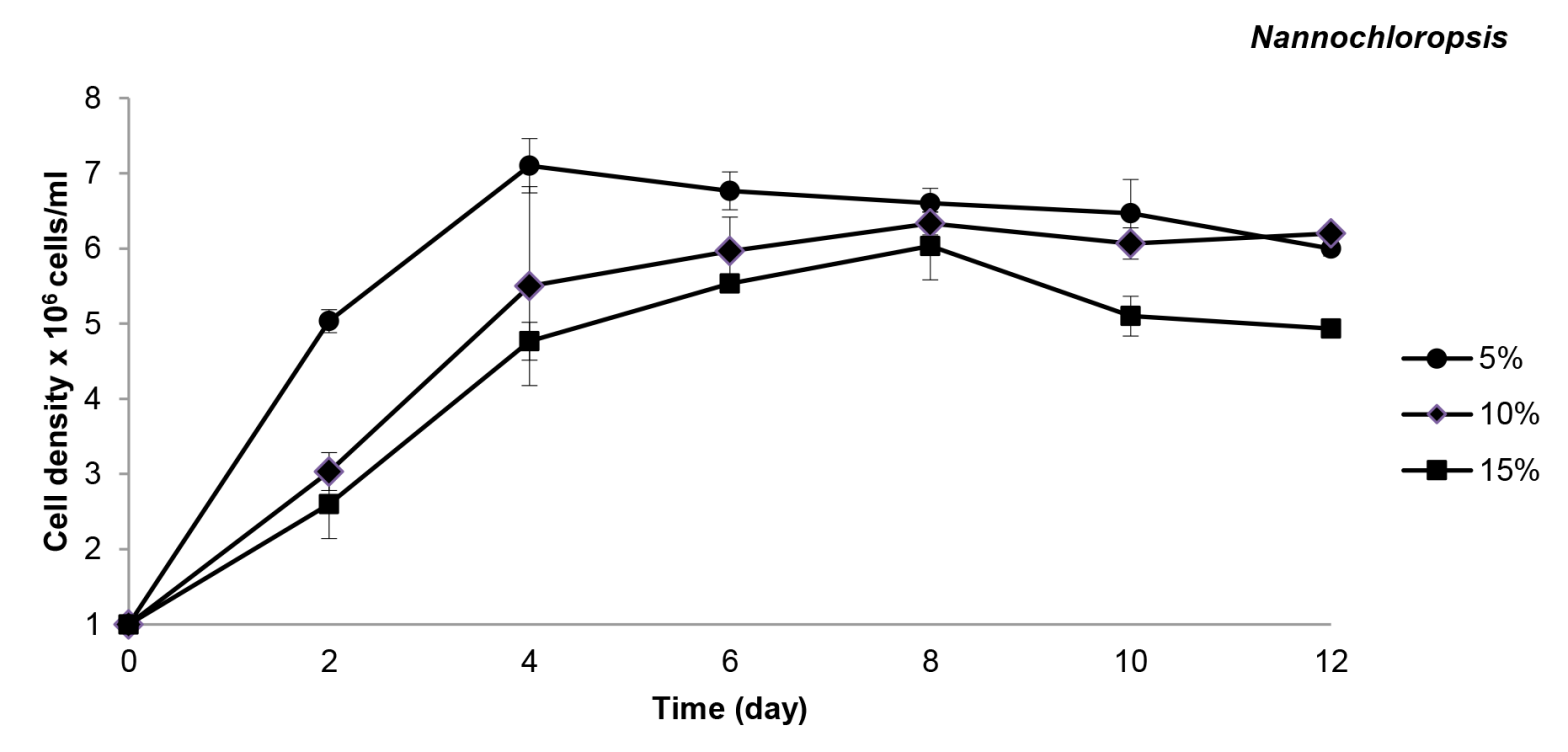
Cell density (Mean ± SD) of *Nannochloropsis* in 5%, 10% and 15% of leachate concentrations.

**Figure 2:**
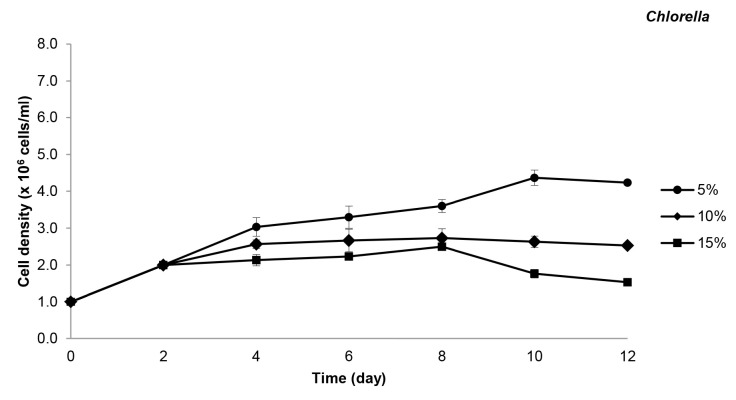
Cell density (Mean ± SD) of *Chlorella* in 5%, 10% and 15% of leachate concentrations.

In the experiment, the cell densities of both species tested were significantly decreased with the increasing concentration (decreasing dilutions) of the leachate. The highest cell density was recorded in the 5% diluted leachate, followed by 10%, and the lowest cell density was observed in 15% diluted leachate. Therefore, both *Chlorella* and *Nannochloropsis* showed significantly higher cell density in 5% diluted leachate compared to 10% and 15% (*p* < 0.05) throughout the 12 days of cultivation (Figs. 1 and 2).

*Nannochloropsis* showed good survival ability in the diluted leachate. The cell density of *Nannochloropsis* is sharply enhanced, depicted by faster cell multiplication and higher cell growth in 5% diluted leachate (Fig. 1). The highest cell density at this concentration is 7.1 ± 0.4 × 10^6^ cells/mL, which can be seen on the fourth day. However, *Nannochloropsis* cultured at higher leachate concentrations, i.e., 10% and 15%, exhibited lower cell density and slower growth, and throughout the study, they reached the highest cell densities of 6.3 ± 0.2 × 10^6^ cells/mL and 6.0 ± 0.5 × 10^6^ cells/mL on day 8, respectively. On the contrary, although *Chlorella* could also survive in all leachate concentrations, it exhibited slow increment of cell density in all three leachate concentrations compared to *Nannochloropsis*. For the first two days, *Chlorella* only reached the highest cell density of 2.0 ± 0.0 × 10^6^ cells/mL in all leachate concentrations. After the second day, the cell density of *Chlorella* in 5% leachate showed a more active cell multiplication and demonstrated a slight enhancement in cell density (Fig. 2); leading to continuous increase of their growth until it reached the highest cell density of 4.4 ± 0.2 × 10^6^ cells/mL on day 10. Nevertheless, in the higher leachate concentrations, the cell density showed a slower increment until the end of the experiment. The highest *Chlorella* cell density recorded for 10% and 15% leachate were 2.7 ± 0.3 × 10^6^ cells/mL and 2.5 ± 0.1 × 10^6^ cells/mL, respectively.

**Figure 3:**
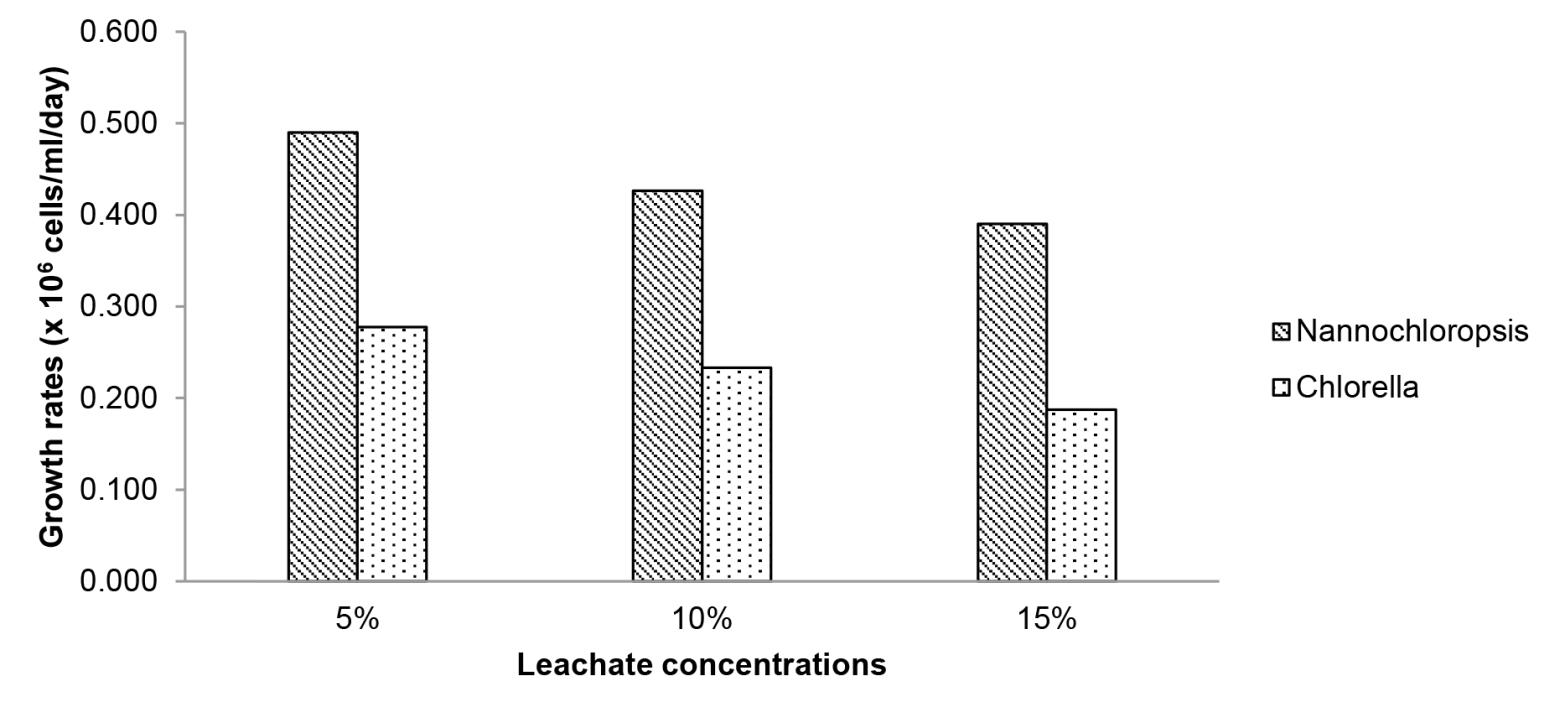
Growth rates of *Nannochloropsis* and *Chlorella* in 5%, 10% and 15% of leachate concentrations.

In terms of growth rate, the average daily growth rates of *Nannochloropsis* and *Chlorella* were significantly different between the leachate concentrations tested (*p* < 0.05). For both species, the highest growth rate was observed in 5% leachate, followed by 10%, and the lowest growth rate was in the 15% leachate. However, between these two species, the growth rate of *Nannochloropsis* was significantly higher compared to the growth rate of *Chlorella* in all leachate concentrations (Fig. 3). Even though all leachate concentrations supported the growth of *Nannochloropsis*, the growth rate was augmented only in the lower leachate concentration and suppressed in the higher concentrations. The 15% leachate was discovered to be unfavourable to the growth of both *Nannochloropsis* and *Chlorella*.

Other than the cell growth, changes of COD and nutrients of the microalgal culture were also observed. After 12 days, the concentrations of COD, NH_3_-N, and PO_4_^3−^ in the control group did not change but were significantly (*p* < 0.05) reduced in both microalgal cultures. In other words, the concentrations of COD, NH_3_-N, and PO_4_^3−^ in the control groups (no microalgae inside) tested did not show much change of nutrient content at the end of the study compared to the microalgal group. The changes or reduction of COD nutrient were related to the removal ability of both microalgae species. The value changes of COD, NH_3_-N, and PO_4_^3−^ content are shown in Table 2. Among the three concentrations tested, 5% leachate showed a significantly high COD reduction from the microalgal culture of both *Chlorella* and *Nannochloropsis*, whereby the concentration dropped from 2500 mg/L to 465 mg/L and 1460 mg/L, respectively. The reduction of COD in leachates were associated with the COD removal efficiency of *Chlorella* and *Nannochloropsis*. These values indicated that nearly 82% of COD was removed from the *Chlorella* culture, and 42% of COD was removed from the *Nannochloropsis* culture in 5% leachate. Meanwhile, the concentration of COD in the 10% leachate decreased from 3500 mg/L to 2807 mg/L and 2535 mg/L, while the similar reduction is also observed in the 15% leachate, from 4500 mg/L to 4200 mg/L and 4097 mg/L, respectively (Table 2).

NH_3_-N was significantly removed by *Chlorella*, where the concentration dropped from 150 mg/L to 25 mg/L in 5% leachate, 250 mg/L to 92 mg/L in 10% leachate, and 350 mg/L to 155 mg/L in 15% leachate. In total, about 83.3% of NH_3_-N was removed by the end of the study in 5% leachate. PO_4_^3−^ was drastically reduced in the diluted leachates containing microalgae compared to the control. During the 12-day experiment, *Chlorella* removed about 94% of PO_4_^3−^ in 5% leachate, 95% of PO_4_^3−^ in 10% leachate, and 86.7% of PO_4_^3−^ in 15% leachate. *Nannochloropsis* showed a slightly lower PO_4_^3−^ removal with a total of 74% in 5% leachate and 77% in 10% leachate compared to *Chlorella*, but the same amount of 86.7% in 15% leachate (Table 2).

## DISCUSSION

The initial concentrations of COD and NH_3_-N in the leachates used in this study were in accord with the findings by Norlailatul *et al*. (2005). The authors, who carried out a study at Air Hitam Sanitary Landfill, Puchong, Selangor, Malaysia revealed that COD reading for leachate varied from 1212 mg/L to 4443.2 mg/L, while NH_3_-N content might reach up to 419.17 mg/L. The initial concentration of COD and NH_3_-N in the present study is also supported by Kamaruddin *et al*. (2013) and Khai and Trang (2012). The research study by Kamaruddin *et al*. (2013) reported that COD and NH_3_-N concentration might reach as high as 1365 mg/L and 1322 mg/L, respectively, and this usually can be seen on old or stabilised leachate (>20 years old pond). Meanwhile, according to Khai and Trang (2012), due to the complexity of leachate composition, COD values may vary from 500 mg/L to 10000 mg/L, and the landfill leachate may contain between 200 mg/L to 1000 mg/L NH_3_-N. COD and NH_3_-N content of the leachate mainly depends on the application of aeration and the age of the landfill itself.

The safe level outlined by the Department of Environment Malaysia (2010) for sewage discharge are 400 mg/L COD, 5 mg/L NH_3_-N and ≤ 10 mg/L PO_4_^3−^ to any inland waters or Malaysian waters. High COD and nutrient concentrations in leachate will bring a significant environmental issue upon leachate leakage into waterways since a large number of toxins will end up in the rivers, lakes, reservoirs, estuaries and coastal areas. Hence, the authorities should promptly take necessary action to adopt new approaches or technologies to improve treatment and disposal processes of landfill leachate.

To address the issue of leachate contamination in coastal areas, a toxicity evaluation of leachate in the areas can be done using marine microalgae as test species. These species acted as the indicator of a sustainable ecosystem. The ability of microalgae to grow in different leachate concentration was studied at the laboratory scale. *Chlorella* showed a slow increment in growth compared to *Nannochloropsis*, and this might be related to NH_3_-N concentration (Cheng & Tian 2013). The content of NH_3_-N was believed to cause a toxic effect on *Chlorella*. Kallqvist and Svenson (2003) also pointed out that high levels of NH_3_-N were the prime cause of toxicity that leads to growth inhibition. Furthermore, the growth inhibition of *Chlorella* was substantially proportional to NH_3_-N concentrations tested. The results of cell density during the experimental period indicated that *Chlorella* is less tolerant to higher leachate concentration, especially in 15% leachate, which contained initial NH_3_-N of 350 mg/L compared to *Nannochloropsis*. This NH_3_-N concentration might pose a toxic effect on *Chlorella*; hence, portray intolerable to the high concentration of leachate.

Although the ability to grow in leachate is limited for both microalgae, especially in high leachate concentration, these two species showed the potential to remove nutrient from leachate in the lower concentration of leachate. The change or reduction in nutrients, e.g. NH_3_-N and PO_4_^3−^ content in leachates is believed to be related with the removal ability by *Chlorella* and *Nannochloropsis*, since Marchao *et al*. (2017) reported that microalgae had been used to remove nitrogen, phosphorus and COD from various types of effluents, because of their adaptability of growing in various wastewater streams. However, different leachate concentration and microalgae species might be the reason for the different removal efficiencies. Cheng and Tian (2013) and Huang *et al*. (2002) reported COD removal of 37.1% and 71.6%, respectively, in their study and microalgae showed nutrient removal ability by utilising those nutrients to grow. Microalgae can convert those nutrient and necessities into energy and use that in cell development. According to He and Xue (2010), the removal of NH_3_-N was mainly attributed to its utilisation by the microalgae to synthesise new biomass under light condition. Besides, microalgae are also useful in carbon dioxide fixation process (Doria *et al*. 2012). This statement is supported by Przytocka-Jusiak *et al*. (1984), who reported that microalgae utilise NH_3_-N as the nitrogen source. The result of NH_3_-N removal from this study was comparable to the study by Huang *et al*. (2002), who described the adoption of *Chorella pyrenoidosa* to purify the landfill leachate and a study by Cheng and Tian (2013) about landfill leachate treatment using *Scenedesmus* sp. Both studies reported that the removal efficiency of NH_3_-N using microalgae in leachate was 76.1% and 72%, respectively. However, the removal efficiency of PO_4_^3−^ in both microalgae were comparable, i.e., in the range of 80.5%–91%, as reported by Cheng and Tian (2013), who stated that the loss of PO_4_^3−^ was attributed to algal metabolic uptake. These preliminary results demonstrated the feasibility of using a cost-effective growth medium, i.e. leachate for algal biomass. The microalgae could be used as a more practical approach for landfill leachate treatment.

Besides, there is a possibility of using these microalgae in other wastewater treatment, i.e., agro-industrial waste such as palm oil mill effluent (POME). The pollution level of agro-industrial wastewater is measured by their COD value, which usually characterised by high COD of over 70000 mg/L (Wong *et al*. 2013).

## CONCLUSIONS

Both microalgal species exhibited similar growth trend in different concentrations of leachate, i.e., 5% leachate enhanced their growth, while leachate at higher concentrations, i.e. 10% and 15% reduced the growth of microalgae as early as at the beginning of the test. In terms of growth rate, *Nannochloropsis* showed significantly higher growth compared to *Chlorella* in all leachate concentrations tested. However, from the changes of nutrients observed in this study, it was found that both species exhibited an ability to remove COD and excessive nutrients, such as NH_3_-N and PO_4_^3−^ in low leachate concentration. Thus, there is the feasibility of using these species for leachate treatment because this approach is a cost-effective, but the appropriate dilution in pre-treatment may be necessary. Some studies reported the potential use of microalgae in bioremediation of wastewater, including industrial waste and palm oil mill effluent (POME) due to their feasibility to reduce pollutant. However, more investigations need to be conducted to explore the ability of *Chlorella* or *Nannochloropsis* in reducing pollutant in leachate or other wastewater treatment. Any further research development should necessarily include the screening of higher tolerant marine microalgae strains since the study on toxicity effect of landfill leachate towards marine microalgae is still lacking.

## Figures and Tables

**Table 1:** Chemical characteristics of diluted leachates.

Parameter concentration (%)	NH_3_-N (mg/L)	PO_4_^3−^ (mg/L)	COD (mg/L)
5	150 ± 0	0.5 ± 0	2500 ± 0
10	250 ± 0	1.0 ± 0	3500 ± 0
15	350 ± 0	1.5 ± 0	4500 ± 0

**Table 2:** Change of COD, NH_3_-N and PO_4_^3−^ content (Mean ± SD) in microalgae culture and in control (no microalgae inside) in different leachate concentrations.

COD/Nutrient	Leachate conc. treatment (%)	Average concentration						

		Initial conc. (mg/L)	Microalgae				Control	

			*Chlorella*		*Nannochloropsis*			

			Final conc. (mg/L)	Reduction (%)	Final conc. (mg/L)	Reduction (%)	Final conc. (mg/L)	Reduction (%)
COD	5	2500 ± 0	456 ± 98	81.8	1460 ± 60	41.6	1714 ± 25	31.4
	10	3500 ± 0	2807 ± 31	19.8	2535 ± 36	27.6	2379 ± 88	32.0
	15	4500 ± 0	4200 ± 100	6.7	4097 ± 119	9.0	4317 ± 180	4.1
NH_3_-N	5	150 ± 0	25 ± 5	83.3	73 ± 3	51.3	112 ± 13	25.3
	10	250 ± 0	92 ± 18	63.2	108 ± 9	56.8	192 ± 18	23.2
	15	350 ± 0	155 ± 9	55.7	222 ± 3	36.6	283 ± 18	19.1
PO_4_^3−^	5	0.5 ± 0	0.03 ± 0.02	94	0.13 ± 0.06	74	0.33 ± 0.06	34
	10	1.0 ± 0	0.05 ± 0.01	95	0.23 ± 0.06	77	0.50 ± 0.20	50
	15	1.5 ± 0	0.2 ± 0.1	86.7	0.2 ± 0	86.7	0.7 ± 0.3	53.3
